# Institutional Notes and News

**Published:** 1911-01-07

**Authors:** 


### Institutional Notes and News.

**Charing Cross Hospital.**

We are glad to remark the good progress which Lady Juliet Duff's Fund on behalf of the Charing Cross Hospital continues to make. Certainly the efforts being made on its behalf are varied and enterprising. An appeal committee, which is supported by the most distinguished names in London society, has been formed, and at the same time Lady Wantage has fulfilled her promise, her donation of £1,000 having been received. The New Year therefore sees the Charing Cross Hospital appeal supported vigorously, and we may mention that the theatrical profession has come forward, and on Wednesday, January 11, Miss Andrews will produce Ibsen's *Ghosts* at the Hôtel Cecil, the time of the performance to be announced later. Those supporters of the hospital who happen to be connoisseurs of the drama will notice the delightful nemesis with which the most-abused play, not merely of the nineties, but of the nineteenth century, is now being produced on behalf of a famous hospital. This is all as it should be, and the most cursory observer of the history of modern drama will congratulate the Charing Cross Hospital not merely on the famous play which is being acted in its interests, but on the fact that Miss Janet Achurch, who made the part of Mrs. Alving famous in England, is again to appear in that rôle. Oswald will be played by Mr. Courtenay Thorpe. London has not had the opportunity of seeing Ibsen's *Ghosts* for about five years, and we are happy to find the cause of charity joining hands with genuine modern drama, to what is bound to prove the advantage of both. The public in general will not miss the fact that the Charing Cross Hospital appeal is being furthered in ways that are likely to attract the support of more than one section of the community.

**The Cardiff Infirmary Appeal.**

In our issue published on Christmas Eve we expressed the hope that the public in Cardiff would not allow its Christmas holidays to interfere with the active prosecution of the appeal on behalf of Cardiff Infirmary. By a happy coincidence, the Cardiff newspapers of the very same day contained as their most important contribution a letter of two columns, signed by Colonel E. M. Bruce Vaughan, urging the infirmary's claims. These were put forward in a reasoned argument, which is a great improvement on the old-fashioned sort of appeal, and which, we understand, has made already a deep impression on the great trading city. At any rate, the appeal has jumped up to £10,000 in three weeks. It has been issued by General Lee, the hospital's much-respected Chairman, and the Colonel's letter is written in its support. After noting the two objects of the appeal, the payment of the infirmary's debt and the increase of its invested capital, a really remarkable phalanx of arguments is brought into line. Among them we note the emphasis laid on the fact that the Newport Road buildings, though built, are built with locked doors till the sum for their upkeep is subscribed, the key being still in the public's pocket. Again, the commercial asset which a spending institution like the great infirmary is to Cardiff is insisted upon, as customer, ratepayer, and universal consumer for the benefit of the sick and the trade of the city. In other words, Cardiff Infirmary spends £15,000 a year in the locality. Finally, we read, the infirmary's claims are not far to seek. "They lie at your very doors. You are in debt to the infirmary to the extent of £21,000 for services already rendered—services for which you have not paid a penny." We have quoted enough to show that this appeal is being prosecuted with originality, and, indeed, the infirmary's case makes really interesting reading. We congratulate General Lee and his colleague on the results they have achieved up to the present, and are sure that Cardiff will look back with gratitude and pride to the day on which it decided to associate its own great charity with the National Memorial of Wales.

**General Hospital, Yarmouth.**

Just before Christmas the half-yearly meeting of the Yarmouth General Hospital was held, and a total debt was revealed of £650, an accumulation of two years, to which attention was drawn by the hon. secretary, Mr. R. F. E. Ferrier. The question of the cost and payment of the children's ward is becoming urgent, for, while it is feared that the cost may amount to more than £400, at present only £150 has been received in subscriptions. Where is the necessary £300 to complete the payment to come from? We feel sure that the hospital's supporters in Yarmouth—and, indeed, the public in general—have only to consider Dr. R. H. Shaw's words on the economy with which the institution is managed, on the more complicated and serious work which he remarked that the hospital is now doing, to come forward and start the New Year with their hospital freed from debt. We hope that the clergy did not fail to couple with the reminder that Christmas is often called the children's festival the fact that the children's ward at the Yarmouth General Hospital is in serious need of subscriptions. The furnishing of this ward has been paid for entirely by Mr. H. P. Fenner. It should not be too much to ask the public of Yarmouth to follow his generous example.

**Bridgwater Hospital.**

The medical report of the Bridgwater Hospital has some interesting facts to notice on the vexed question of recommendations. A new system was tried, we believe a year ago, and the result is seen to be an important decrease in the number of out-patients, though it is hardly surprising to learn that they are still 300 more in number than they were in the year 1905, when the hospital first became free. Hospital construction at Bridgwater has taken the form of a new hot-water system, an entirely renovated operation theatre, and an installation of electric light, the latter being a present from Mr. E. Jardine. The in-patients numbered 568; while an analysis of the numbers treated in the out-patient department shows 823 to have been admitted, exclusive of 521 renewals; or, in other words, a total number of out-patients amounting to 1,344. Annual subscribers here, again, show a slight tendency to diminish in numbers, which is unfortunate in that there is an accumulated debt of £558. The Bridgwater Hospital is approaching the year of its centenary; it is, in fact, ninety-seven years old, and its supporters will applaud the scheme of reconstruction which the institution's hon. architect (Mr. A. B. Cottam) is preparing, in the hope that the public's generosity will provide the means for their hospital to start its second hundred years properly equipped for the new century.

**Coventry and Warwickshire Hospital.**

Rear-Admiral Bacon, who presided the other day at the annual meeting of the Coventry and Warwickshire Hospital, made a stimulating speech, from which we take the following passages: Both the number of in-patients and out-patients had increased considerably. They also had to report the fact that the actual economy, largely due to the supervision of Miss Barker, the matron, had been increased this year. Another matter equally important was the number of complaints received with respect to the treatment of patients. He had been at some trouble to ascertain from Mr. J. A. Rudd, the secretary, exactly what complaints had been received during the past year, and he found they numbered four. On investigation three of them, he was informed, could not stand. In the case of the fourth, there was a certain amount of grumbling, but he thought they would agree with him that one complaint substantiated in a hospital of that sort, which dealt with a large number of in-patients and out-patients, was an extremely good record. As to whether the hospital was advancing on up-to-date lines, Admiral Bacon said they had only to look at that excellent out-patient department (in which the meeting was being held) to dispel any doubts on that subject. Turning to the financial position, they would notice that there was a balance of £311 out of a total income of £6,608, and that so far was satisfactory. It was very encouraging to notice that the King Edward Extension Fund, which had been started in memory of King Edward, would supplement the hospital accommodation by an additional wing, and thus, it was hoped, would accommodate extra patients, and at the same time prevent the overcrowding that at present necessarily took place. Rear-Admiral Bacon noticed that on the agenda for that meeting there was no mention made of one person, and one person to whom he thought the hospital owed a very great debt both as regarded munificence of subscription and also for the time devoted to the hospital. He alluded to Mr. Alfred Herbert, the chairman of the general committee. Although he (the chairman) knew that he was absolutely out of order in mentioning this, still he felt that he was merely voicing the feelings of thousands of patients in that hospital when he said that they owed a very great debt of gratitude to Mr. Herbert for the time and energy he devoted to the institution. Mr. Herbert made an interesting speech in seconding the adoption of the report, in which he gave the two principal objections he held against rate-supported institutions. First, he thought the patient would lose the feeling of hospitality he now had on entering, and might even feel the stigma of the Poor-law; and, secondly, because the support of a voluntary hospital offered a field for social work, apart from creed or party, that the cause of good citizenship could not afford to lose. [We regret that we have no space to quote further from a really able speech.]

**Leicester Infirmary.**

The Christmas festivities of 1910 will long be remembered by the 260 patients in the Leicester Infirmary. On Christmas Day special fare was provided for the patients, turkey and roast beef and Christmas pudding; and visiting was permitted in the afternoon. The nurses' carol party paid a round of visits to the wards at 5 A.M., and each patient was given a present. On Boxing Day visiting was again permitted, and on this and the following day the ward tea-parties to patients and their friends took place. On Wednesday the children's Christmas tree and entertainment was given in the out-patients' hall. The tree was 27 feet in height, and was lighted with numerous electric fairy lights and decked with presents of all kinds. On Thursday Mr. T. Crew's concert party entertained the patients in the accident ward, on Friday the household servants' dinner was given, on Tuesday last the nursing staff gave a delightful entertainment in the Odames ward, on Wednesday the nursing staff held their invitation dance, and on Thursday Mrs. F. W. Forester (the wife of the master of the Quorn) kindly engaged a party of entertainers for the patients and assembled guests in the Oliver ward, giving a splendid finish to the festivities. Miss Rogers, the lady superintendent, is to be congratulated heartily on the excellent cycle of arrangements which she organised.

**The Queen Visits Lynn Hospital.**

Surprise visits by Royalty to hospitals are always memorable, particularly to the junior members of the staffs, who often have at such times an opportunity not always forthcoming on more formal occasions. On Tuesday last the Queen, accompanied by the Prince of Wales, Prince Albert, and Princess Mary, drove from Sandringham and paid an unexpected visit to the West Norfolk and Lynn Hospital. In the absence of the matron (Miss Swain), her Majesty was received by Miss Austin, the deputy-matron, and by the house surgeon, Mr. Richard Charles. Sir Somerville Gurney, who was called by telephone, attended her Majesty round the wards. The Queen came specially to see a little boy named Sidney Barker, who had written to her Majesty thanking her for the toys and books sent by her at Christmas, and asking her to come and see the boys. Her Majesty, on arriving at Sidney Barker's cot, said, "You see, I have come as you asked me to do." She also showed particular interest in a little boy who had a broken ankle, but we understand that all the children were too shy to reply to any of the remarks the Queen addressed to them. The visit lasted about half an hour, and the Royal visitors all signed the minute-book.

